# How Reproducible Are the Ultrasound Features of Adenomyosis Defined by the Revised MUSA Consensus?

**DOI:** 10.3390/jcm14020456

**Published:** 2025-01-13

**Authors:** Nikit Kadam, Somia Khalid, Kanna Jayaprakasan

**Affiliations:** 1 Derby Fertility Unit, Royal Derby Hospital, University Hospital of Derby and Burton, Derby DE22 3NE, UK; 2 Faculty of Medicine and Health Sciences, University of Nottingham, Nottingham NG7 2RD, UK

**Keywords:** adenomyosis, MUSA criteria, 3D ultrasound, inter-observer reproducibility, intra-observer reproducibility

## Abstract

**Background/Objectives**: The aim of this study is to assess the inter- and intra-observer reproducibility of the identification of direct and indirect ultrasonographic features of adenomyosis as defined by the revised Morphological Uterus Sonographic Assessment (MUSA) consensus (2022). **Methods**: A cohort of 74 women, aged 18 to 45, were recruited from the recurrent miscarriage and general gynaecology clinic at a university-based fertility centre. All the participants underwent 2D and 3D transvaginal Ultrasound scan (TVS) examination in the late follicular and early luteal phase. Conventional grey scale and power Doppler image volumes were acquired and stored. Subsequently, the stored 3D ultrasound images were independently re-evaluated offline by the two observers for the direct and indirect features of adenomyosis as outlined by the revised MUSA group. The intra- and the inter-observer reproducibility was estimated using Cohen’s Kappa coefficient. **Results:** The intra- and interobserver reproducibility (K −0.27, 95% CI 0.06–0.48 and K 0.13, 95% CI −0.10–0.37, respectively) for at least one direct feature of adenomyosis was only modest. Amongst the individual direct features, the interobserver variability of identifying myometrial cysts was fair (K 0.21, 95% CI −0.00–0.42), whereas the intra-observer variability was moderate (K 0.44, 95% CI 0.26–0.63). While hyperechogenic islands identification achieved a fair level of intra- (K 0.31, 95% CI 0.09–0.53) and interobserver (K 0.24, 95% CI 0.01–0.47) agreement, the reproducibility of reporting sub-endometrial lines/buds was fair for the intra-observer (K 0.22, 95% CI −0.02 0.47) and poor for the interobserver (K 0.00, 95% CI −0.20–0.19). The interobserver agreement for indirect features varied from poor to moderate, while the intra-observer agreement ranged between poor to good. **Conclusions**: The reporting of adenomyosis using direct features suggested by the revised MUSA group consensus showed only modest interobserver and intra-observer agreement. The definitions of ultrasound features for adenomyosis need further refining to enhance the reliability of diagnosis criteria of adenomyosis.

## 1. Introduction

Adenomyosis, a benign gynaecological condition, is characterised by the presence of endometrial glands and stroma in the myometrium with or without hyperplasia of the surrounding myometrium. While 30% of women with adenomyosis may be asymptomatic [1], they may experience abnormal uterine bleeding, dysmenorrhea, and infertility. The prevalence of adenomyosis varies widely depending on the population studied and the diagnostic tool and criteria used [2,3]. Traditionally, adenomyosis is diagnosed using histology of the hysterectomy specimen, but transvaginal ultrasound scan (TVS) and magnetic resonance imaging (MRI) are the commonly used non-invasive diagnostic modalities. Whilst the diagnostic accuracy of TVS and MRI is comparable [4], TVS is often the favoured first-line imaging modality.

Since Walsh et al. reported ‘sonolucent areas’ and ‘speckled echo-pattern’ as the ultrasound diagnostic features of adenomyosis [5], many ultrasound features of adenomyosis have been described [2,6,7]. In 2015, the international Morphological Uterus Sonographic Assessment (MUSA) group [8] published a consensus statement which proposed standardised terminology and criteria for ultrasound features of adenomyosis to facilitate uniform reporting in clinical practice and research.

Whilst the diagnostic approach using MUSA criteria has standardised the terms and may have potentially enhanced the diagnostic accuracy of adenomyosis, the diagnostic criteria used should be reproducible between users with different levels of experience. Rasmussen et al. reported low- to moderate-level agreement for diagnosing adenomyosis even amongst experienced practitioners [9,10]. The limited intra- and interobserver agreement of the original MUSA criteria may be related to unclearly defined features. In 2022, using a modified Delphi procedure, the MUSA group redefined the features and classified them into direct and indirect signs of adenomyosis [11]. The reproducibility studies on the redefined MUSA features have not yet been reported or published. Therefore, we performed this study to evaluate the intra-observer and interobserver agreement of the diagnosis of adenomyosis using the direct and indirect ultrasound features defined by the revised MUSA consensus.

## 2. Materials and Methods

### 2.1. Study Design

In this prospective observational study, a total of 74 consecutive women, aged between 18 and 45, seen in the recurrent miscarriage clinic and general gynaecology clinic, were recruited. Of the 74 women participating in the study, 69 had experienced recurrent miscarriages, and five were healthy fertile women. All participants were asymptomatic at the time of recruitment. The women with recurrent miscarriages were not on any hormonal treatments, whereas the healthy fertile women were using contraception. Women with BMI exceeding 40 kg/m^2^ were excluded from the study. The study received a formal approval from the local Ethics committee. Written informed consent was gained from the participants. All participants underwent 2D and 3D transvaginal ultrasound examinations in the late follicular phase or early luteal phase of their menstrual cycles.

### 2.2. Ultrasound Data Acquisition

The ultrasound scan was conducted by a single investigator (NK) using the Voluson Swift BT 23 ultrasound machine (GE Healthcare, Chicago, IL, USA), which was equipped with a 5–9 MHz 3D transvaginal probe. Each participant was asked to empty their bladder and was scanned supine in a modified Lloyd-Davies position that ensured participants were comfortable and the transvaginal transducer could be manipulated freely. A conventional ultrasound (2D) evaluation of uterus and ovaries were initially performed. For 3D ultrasound volume acquisition, the uterus was initially visualised in mid-sagittal plane displaying both endometrial cavity and cervical canal simultaneously. Using the 3D volume sector, to define the region of interest, the volume box was expanded to encompass the uterus and cervix. The sweep angle was then set to 120° to include the entire uterus. Subsequently, 3D volume of the uterus was obtained with sweep mode set at high quality slow-sweep setting. Additionally, to minimise movement artefact and enhance image quality, the probe was held steady, and participants were instructed to remain still during the acquisition process. The acquired 3D volume was examined in multiplanar view to ensure the entire uterus had been captured. If the acquired 3D volume data were not displaying the uterus adequately or if the image quality was poor, the scan was repeated to obtain a satisfactory 3D volume dataset. Three-dimensional Power Doppler imaging was used to assess the vascularity of the uterus. The images were stored on the hard drive of the machine to facilitate off-line analysis later.

### 2.3. Ultrasound Data Analysis

**Figure 1:**
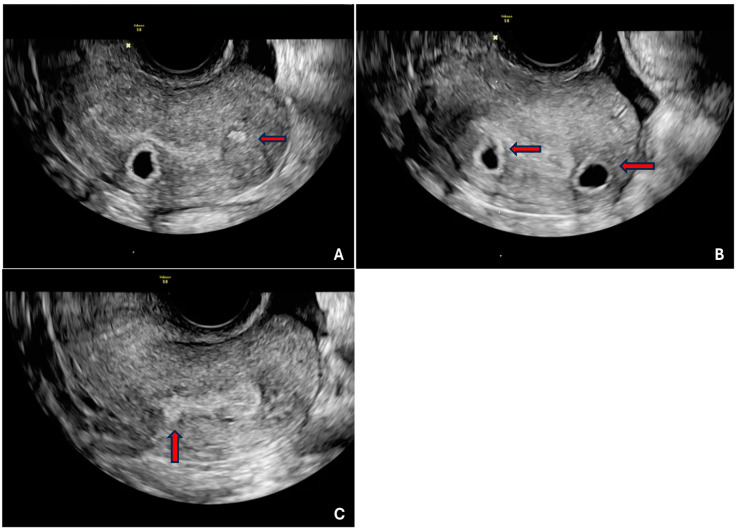
Direct features of adenomyosis—hyperechoic islands (**A**), myometrial cysts (**A**,**B**), and echogenic sub-endometrial buds (**C**). Red arrows are to indicate the hyperechogenic islands, myometrial cyst and echogenic buds.

**Figure 2:**
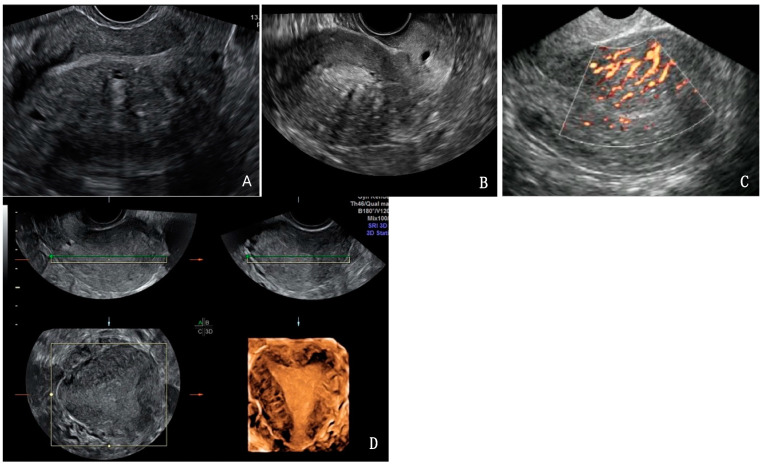
Indirect features of adenomyosis—globular uterus (**A**), asymmetrical myometrium (**A**), fan-shaped shadowing (**B**), trans-lesional vascularity (**C**), irregular junctional zone and interrupted junctional zone (**D**).

Two investigators (NK and SK), both gynaecologists, with over one year of experience in gynaecology ultrasound independently analysed the 3D volume datasets offline on the ultrasound machine and personal computer utilizing dedicated software (GE healthcare 4D view, version 7.0), respectively. The stored 3D volume datasets were re-analysed on the ultrasound machine by one investigator (NK) two months after the initial review to assess intra-observer variability. Both investigators, blinded to each other, independently evaluated each image for the presence or absence of direct (myometrial cysts, hyperechoic islands, sub-endometrial lines, or buds) (Figure 1) and indirect features (globular uterus, asymmetrical thickening, fan-shaped shadowing, trans-lesional vascularity, irregular junctional zone, interrupted junctional zone) (Figure 2), as outlined in the revised MUSA consensus and documented on a case report file. The dataset was opened, initially displayed in the multiplanar view, and the user was able to scroll through one image plane at a time. One image plane (longitudinal, transverse, or coronal) could be selected to view entirely on the screen to assess the image closely for the features of adenomyosis. To facilitate accurate evaluation of the structure or point of interest, a reference dot (multiplanar axis dot) was viewed in each orthogonal plane. This placement allowed precise differentiation between myometrial cysts and the vascular component while navigating through the images. The 3D volume datasets were also systematically rotated and analysed across three axes (X, Y, Z) to precisely identify sub-endometrial lines and buds, as well as to assess interruptions and irregularities within the junctional zone. In instances of suspected asymmetrical myometrial thickening, measurements of the anterior and posterior uterine walls were obtained in the longitudinal plane. A difference of greater than 5 mm between these measurements was classified as asymmetrical [11]. The diagnosis of adenomyosis was established by the presence of at least one direct feature on ultrasound examination of the images. A study checklist was employed to guarantee thorough and systematic data collection. The quality of the images was assessed and categorised into three distinct levels as optimal—images that met the highest standards of clarity and detail, providing excellent information of ultrasound features; sub-optimal—images that met satisfactory standards but contained minor imperfections or lacked certain details; poor—images that did not meet the acceptable quality standards, exhibiting image degradation or distortion hindering data collection. Only optimal images were included in the reproducibility study.

### 2.4. Statistical Analysis

A statistical analysis was performed using SPSS software (Version 29). Given that the collected data were nominal, the agreement between the two investigators was assessed by calculating the Cohen’s Kappa coefficient and a 95% confidence interval. The levels of agreement for the Cohen Kappa coefficient were standardised according to established criteria 0.00–0.20 = Slight/Poor agreement, 0.21–0.40 = fair agreement, 0.41–0.60 = Moderate, 0.61–0.80 = good agreement, 0.81–1.00 = Very good agreement. Observed and expected agreement calculations were also undertaken. Observed agreement represents percentage of agreement between two observers while expected agreement refers to percentage of agreement that is likely to occur purely by chance.

The outcome measures assessed were inter- and intra-observer reproducibility for direct and indirect ultrasound features of adenomyosis as measured using Cohen Kappa coefficient. The direct features assessed were myometrial cysts, hyperechoic islands, sub-endometrial lines or buds. The indirect ultrasound features evaluated were globular uterus, asymmetrical thickening, fan shaped shadowing, trans-lesional vascularity, irregular junctional zone, interrupted junctional zone.

## 3. Results

Of the seventy-four datasets, a total of sixty-eight were analysed in this study. Six datasets were excluded due to suboptimal or poor image quality (*n* = 3), the presence of fibroids (*n* = 2), and axial positioning of the uterus (*n* = 1), which hindered the assessment of relevant ultrasound features. The mean (±standard deviation) age of the participants was 34.2 ± 5.7 years. In total, 75% of the participants had an anteverted uterus. Reviewer 1 identified at least one direct feature of adenomyosis in fifty-three datasets, while Reviewer 2 reported such features in 38 datasets, yielding prevalence rates of 78% and 56%, respectively (Table 1).

### 3.1. Interobserver Agreement

The diagnosis of adenomyosis based on the presence of a single direct feature achieved a fair level of agreement between the two observers, indicated by a Kappa statistic of 0.28 (95% CI 0.06–0.48). The observed and expected diagnostic agreement for adenomyosis was 66.1% and 53.2%, respectively. Among the direct features assessed, both myometrial cysts and hyperechogenic islands exhibited a fair level of agreement, with Kappa coefficients of 0.21 (95% CI −0.00–0.42) and 0.25 (95% CI 0.01–0.47), respectively. However, sub-endometrial lines and buds demonstrated a poor level of agreement (Table 2).

In relation to the indirect features, the agreement varied from poor to moderate (Table 2). The most reproducible indirect feature identified was a globular uterus, which had a Kappa coefficient of 0.55, while the least reproducible feature was fan-shaped shadowing with a Kappa coefficient of 0.20 (95% CI 0.04–0.35).

### 3.2. Intra-Observer Agreement

The prevalence of adenomyosis after the first review and second review was 78% and 68%, respectively, with at least one direct feature identified in 53 datasets on the first review and 47 datasets after the second review (Table 3).

The intra-observer agreement for diagnosis of adenomyosis based on at least one direct feature exhibited poor reproducibility (K = 0.13, 95% CI −0.10–0.37). Among the individual direct features, both hyperechogenic islands and sub-endometrial lines/buds reached fair level of agreement, while myometrial cyst achieved a moderate level of agreement with Kappa coefficient of 0.45 (95% CI 0.26–0.63). The agreement levels for indirect features ranged from poor to good, with globular uterus and asymmetrical myometrial thickening achieving highest reproducibility, whereas fan-shaped shadowing demonstrated a poor level of agreement (Table 4).

## 4. Discussion

This study evaluated intra- and interobserver agreement in the ultrasound diagnosis of adenomyosis based on the modified MUSA consensus, and it appears that the level of agreement is widely varied. This is the first study reporting the reproducibility of the diagnosis of ultrasound features of adenomyosis proposed by the MUSA consensus, modified in 2022. While the interobserver reproducibility for at least one direct feature was fair with a Kappa statistic of 0.28 (95% CI 0.06–0.48), the intra-observer reproducibility was poor (Kappa = 0.13, 95% CI −0.10–0.37). Amongst the direct features, the interobserver agreement was highest for myometrial cysts (K = 0.21, 95% CI −0.00–0.42) and hyperechogenic islands (K = 0.24, 95% CI 0.01–0.47), whereas sub-endometrial lines and buds demonstrated the lowest level of agreement (K = 0.00, 95% CI −0.20–0.19). Myometrial cyst also achieved a higher level of intra-observer reproducibility, while hyperechogenic islands and sub-endometrial lines and buds reached fair level of variability. The reproducibility of indirect features exhibited a range from poor to moderate levels of interobserver agreement and poor to good levels of intra-observer agreement. Specifically, the feature of a globular uterus demonstrated a good to moderate level of intra- and interobserver reproducibility, while fan-shaped shadowing reflected a slight level of agreement. Furthermore, all the other indirect features, such as asymmetrical myometrial thickening, trans-lesional vascularity, irregular junctional zone (JZ), and interrupted JZ, attained fair to good levels of intra- and interobserver agreement.

Direct features serve as definitive indicators of adenomyosis, which is characterised by the presence of endometrial tissue infiltrating the myometrium. The existing literature on intra- and interobserver variability in the 2D and 3D ultrasound features of adenomyosis is relatively limited, and the findings have been inconsistent. A noteworthy study by Neal et al. [12] assessed interobserver agreement among five reviewers, revealing a fair level of agreement for myometrial cysts, while the agreement regarding hyperechogenic islands was found to be poor. Additionally, it is important to highlight that sub-endometrial lines and buds were excluded from this study. Another study by Andersson et al. [13] highlighted that myometrial cyst had a poor interobserver variability. Conversely, hyperechogenic islands and sub-endometrial lines/buds displayed fair levels of agreement. In our review, myometrial cyst and hyperechogenic islands had fair interobserver agreement, but sub-endometrial lines/buds showed poor reproducibility. The discrepancies in agreement may be attributed to several factors. Small cysts and hyperechogenic islands may be easily overlooked during transvaginal ultrasound (TVUS), leading to inconsistent diagnoses. Conversely, these features may also be considered representative of a typical aspect of uterine architecture. Furthermore, there is a lack of comprehensive studies defining the precise size thresholds of these cysts and islands which could contribute to the difficulties in achieving consistent interpretations. Moreover, the 2022 update from the MUSA group [11] does not offer definitive clarity concerning the definitions and specific size thresholds for cysts and islands that should be included in the analysis. Addressing these issues may enhance diagnostic accuracy and facilitate better management of patients with adenomyosis. Myometrial cysts can be observed in malignant mesenchymal tumours, like uterine sarcomas due to underlying central necrosis [14], and women with such conditions are usually postmenopausal. The cysts in uterine sarcomas are irregular, with other distinguishing features, including irregular tumour mass and abundant intralesional vascularity [15].

In our study, one observer reported a prevalence of adenomyosis, based on the presence of a single direct feature, of 77%, while the second observer noted a prevalence of 55%. Several studies have documented adenomyosis prevalence ranging from 5% to 70% [16,17,18]. The substantial variability in these figures is predominantly due to the absence of standardised diagnostic criteria for adenomyosis. Upson et al. [19] conducted a thorough epidemiological review, which analysed the prevalence of adenomyosis identified in hysterectomy procedures, revealing a considerable range from 8% to 61% over the last 50 years. In contrast, imaging studies, such as ultrasound and magnetic resonance imaging (MRI), indicate a prevalence of approximately 20%. Furthermore, it is essential to acknowledge the viewpoint presented by Weiss et al. [20], who described adenomyosis as a variant, suggesting that it may not be strictly regarded as a disease entity but rather as a condition that exists within a spectrum of related pathologies.

The emergence of three-dimensional transvaginal ultrasound (TVUS) technology has significantly enhanced our capacity to evaluate junctional zone (JZ) abnormalities, previously assessed primarily through magnetic resonance imaging (MRI). In the present study, we investigated alterations within the JZ, specifically focusing on irregularities and interruptions, which demonstrated a fair level of interobserver agreement and fair to moderate intra-observer agreement. This finding stands in contrast to a prior study [9], which reported low reproducibility rates for JZ features due to challenges in visualizing the JZ clearly. Nonetheless, that study did indicate slightly improved interobserver agreement for 3D volumes analysed by expert reviewers. Furthermore, a reproducibility analysis conducted by Neal et al. [12] revealed poor agreement among five reviewers concerning JZ irregularities. In our research, we employed an offline analysis technique of the JZ across three orthogonal planes derived from the acquired 3D volumes, which facilitated a more accurate assessment of the irregularities and interruptions present within the junctional zone.

In our study, the indirect features, which include a globular uterus, asymmetrical myometrial thickening, fan-shaped shadowing, and trans-lesional vascularity, demonstrated a poor to moderate level of interobserver reproducibility and poor to good intra-observer reproducibility. These findings are consistent with a pilot study conducted by Rasmussen et al. [10], which reported good interobserver variability for well-defined lesions, yet indicated lower variability for adenomyosis and individual features. The observed subjective variation may be attributed to the limited consensus regarding specific individual ultrasound characteristics. The reproducibility study conducted by Dueholm et al. [21] also identified a significant level of disagreement concerning ultrasound features and recommended further studies to evaluate interobserver and intra-observer variability.

Diagnostic tools, such as three-dimensional transvaginal ultrasound (3D TVS) and magnetic resonance imaging (MRI), are utilised for the diagnosis of adenomyosis. Tellum et al. [22] demonstrated that there is no statistically significant difference in the diagnostic accuracy of these two imaging modalities, indicating that neither method holds a definitive advantage in the diagnosis of adenomyosis. Two-dimensional ultrasound diagnosis of adenomyosis using conventional diagnostic criteria demonstrates low interobserver agreement for various ultrasound features [23]. MRI diagnosis [21,24] has been found to have a high level of reproducibility using MRI specific criteria for diagnosis. In contrast, a comparative analysis [13] revealed that 2D and 3D ultrasound scan demonstrated a higher level of interobserver agreement compared to MRI in the diagnosis of adenomyosis.

Uterine biometric measurements obtained through ultrasound scans are increasingly being used to diagnose adenomyosis. However, the effectiveness of these measurements in accurately diagnosing the condition remains inconsistent. A study by Raimondo et al. [25] indicated that the anteroposterior diameter of the uterus tends to increase more than the transverse and longitudinal diameters in cases of adenomyosis, but it also reported a low accuracy rate for diagnosing the condition. The study concluded that larger research efforts are needed to evaluate the usefulness of these biometric measurements in clinical practice.

The emergence of artificial intelligence (AI) as a tool in the medical field has significantly advanced early diagnosis and treatment. A systematic review by Shen et al. [26] demonstrated that the performance of AI is on par with that of clinicians. In contrast, a study by Raimondo et al. [27] evaluated the effectiveness of AI in diagnosing adenomyosis and compared it to the performance of skilled ultrasound trainees. The findings revealed that AI performed sub-optimally in comparison to the ultrasound-trained trainees, prompting the authors to recommend further research to assess the role of AI in the diagnosis of adenomyosis.

The strength of the study is its prospective design along with inclusion of a cohort of consecutive participants. This cohort comprised patients who have had recurrent miscarriage alongside those who had successfully given birth. To ensure unbiased analysis, both the observers reviewed the ultrasound images independently and were blinded to each other’s findings. All ultrasound scans in this study were performed by a single observer to ensure a high level of consistency in image acquisition. To the best of our knowledge this is the first study conducted to evaluate the interobserver variability of the diverse ultrasound features of adenomyosis stated in the revised MUSA group consensus. Additionally, this is one of the few studies to utilise both 2D and 3D ultrasonographic criteria for diagnosing adenomyosis.

One potential limitation of our study is the reliance on offline analysis of datasets and volumes, as opposed to conducting real-time analysis. However, 3D off-line analysis is a comparable model to real-time analysis. It offers the advantage of allowing the reviewer time to assess the images without prolonging the scan time for the patient and away from the stress of a clinical setting. Another limitation of the study is the small sample size, although the numbers included in the study were sufficient to answer the study objective.

## 5. Conclusions

This study demonstrates only a fair level of interobserver and intra-observer reproducibility in the diagnosis of adenomyosis. While the agreement among observers regarding sub-endometrial lines and buds was found to be poor, there was a fair to moderate level of agreement in identifying myometrial cysts and hyperechoic islands. To achieve greater diagnostic accuracy, it is imperative to undertake further investigations that will establish a minimum size threshold for cysts and hyperechoic islands. Further refining of the definitions of the ultrasound features of adenomyosis are needed for more reliable and accurate diagnosis. Definitive diagnosis of adenomyosis using reliable and accurate criteria facilitate appropriate management of patients with adenomyosis leading to improved patient outcomes.

## Figures and Tables

**Table 1:** Distribution of direct features by numbers between the two reviewers.

Direct Features	Reviewer 1 (*n* = 53)	Reviewer 2 (*n* = 38)
One feature	26 (49.1)	17 (44.7)
Two features	22 (41.5)	18 (47.4)
Three features	5 (9.4)	3 (7.9)

Data are given as *n* (%).

**Table 2:** The interobserver agreement with observed and expected agreements in diagnosing adenomyosis and individual ultrasound features.

	Characteristics	Prevalence (n = 68)		Interobserver Agreement (κ)	95% CI	Observed (%)	Expected (%)
		Reviewer 1	Reviewer 2				
	Diagnosis of Adenomyosis (atleast one direct feature)	53 (77.9)	38 (55.8)	0.27	0.06–0.48	66.1	53.2
Direct features	Myometrial cysts	37 (52.8)	25 (35.7)	0.21	−0.00–0.42	60.0	49.1
	Hyperechogenic islands	34 (49.2)	32 (46.3)	0.24	0.01–0.47	62.3	50.0
	Sub-endometrial lines/buds	14 (21)	4 (5.8)	0.00	−0.20–0.19	75.0	75.0
Indirect features	Globular Uterus	7 (10)	16 (22.8)	0.54	0.29–0.79	87.1	71.7
	Asymmetrical thickening	14 (20.2)	28 (40.5)	0.21	0.00–0.43	65.2	55.5
	Fan shaped shadowing	56 (80)	28 (40)	0.19	0.04–0.35	55.7	44.8
	Trans-lesional vascularity	25 (35.7)	17 (24.2)	0.39	0.17–0.62	74.2	57.3
	Irregular JZ	51 (75)	43 (63.2)	0.25	0.02–0.48	67.6	56.6
	Interrupted JZ	27 (39.7)	18 (26.4)	0.31	0.09–0.54	69.1	54.8

Data given as *n* (%). CI indicates confidence interval.

**Table 3:** Distribution of direct features by numbers after first and second review.

Direct Features	First Review(*n* = 53)	Second Review (*n* = 47)
One	26 (49.1)	27 (57.4)
Two	22 (41.5)	15 (31.9)
Three	5 (9.4)	5 (9.4)

Data given as *n* (%).

**Table 4:** The intra-observer agreement with observed and expected agreements in diagnosing adenomyosis and individual ultrasound features.

	Characteristics	Prevalence		Intra-Observer Agreement (κ)	95% CI	Observed (%)	Expected (%)
		First Review	Second Review				
	Diagnosis of adenomyosis (atleast one direct feature)	53 (77.9)	47 (68)	0.13	−0.10–0.37	54	40.7
Direct features	Myometrial cysts	37 (52.8)	21 (29.5)	0.45	0.26–0.63	72	49.1
	Hyperechogenic islands	34 (49.2)	30 (42.3)	0.32	0.09–0.53	66	50.3
	Sub-endometrial lines/buds	14 (21)	21 (30.4)	0.22	−0.02–0.47	70.5	62.1
Indirect features	Globular uterus	7 (10)	5 (7)	0.64	0.30–0.96	94.3	84.4
	Asymmetrical thickening	14 (20.2)	10 (14.1)	0.80	0.61–0.98	94.2	71.4
	Fan-shaped shadowing	56 (80)	53 (73.6)	0.18	−0.07–0.42	70.4	64.1
	Trans-lesional vascularity	25 (35.7)	26 (36.6)	0.36	0.13–0.58	70.4	53.9
	Irregular JZ	51 (75)	40 (57.1)	0.47	0.27–0.67	75.3	53.3
	Interrupted JZ	27 (39.7)	14 (20)	0.38	0.17–0.58	72.4	55.8

Data given as *n* (%). CI indicates confidence interval.

## Data Availability

The raw data supporting the conclusions of this article will be made available by the authors on request.
